# Impact of changes to cervical screening guidelines on age and interval at which women are tested: Population-based study

**DOI:** 10.1177/0969141320953446

**Published:** 2020-08-30

**Authors:** Alejandra Castanon, Shama Sheikh, Philippa Pearmain, Peter Sasieni

**Affiliations:** 1 School of Cancer & Pharmaceutical Sciences, Faculty of Life Sciences & Medicine, King’s College London, London, UK; 2 Public Health England Screening, Screening Quality Assurance Service, Birmingham, UK

**Keywords:** Cervical cancer screening, screening intervals, age at screening, Kaplan–Meier, organised screening

## Abstract

**Background:**

English cervical screening programme guidelines changed between 2009 and 2012. We explore the impact on the age and intervals at which women receive a cytology test.

**Methods:**

Eligible women were controls from a population-based case–control study in England. Tests taken between 1980 and 2017 were extracted from the call/recall database. Using the Kaplan–Meier estimator by birth cohort and age at (or time since) last test, we explore proportions tested since or prior to a given age, years since previous test, and interval following a negative test.

**Results:**

Screening histories from 46,037 women were included. Proportion tested by age 26 has increased from 55% among birth cohorts 1978–1979 to 67% among those born 1990–1991, despite more recent cohorts only having received one invitation (instead of two) prior to age 26. The proportion of women tested at aged 28 with a test three years earlier increased by 20% (from 36% in 1997–2006 to 56% in 2012–2017) whereas the proportion tested at ages 23–27 without a prior test increased from 34% to 80%. The age at last test prior to exiting the programme has decreased: among those born 1928–1931 86% had a test aged 60–65, but only 71% of those born 1947–1951.

**Conclusion:**

Clear programme guidance alongside quality assurance has improved the cervical screening programme by standardising the age and intervals at which women are screened.

## Background

In England, screening using cervical cytology was introduced in 1988 to women aged 20–64. The programme recommended that screening intervals should not be less than three years and no longer than five years, but it was up to each local health authority in place at that time what interval they implemented.^1^ In 2004, the age of first invitation to screening was increased from 20 to 25 years and screening intervals were standardised across the country to be three yearly for women aged 25–49; and five yearly for women aged 50–64. The policy was not retrospective, so that women already invited at age 20 were invited again at age 23. Hence it was not until 2009 that no women were first invited under age 25. In 2012, the age at which the first screening invitation was sent changed once more; this time to 24.5 years (to enable women to be screened by their 25th birthday). As of 2012, triage of low-grade cytology using Human Papillomavirus (HPV) testing was rolled out in England.^2^ Full rollout was completed in 2013. Such triage has the advantage of eliminating early recall (i.e. returning for screening in a year) by either referring those postive immediately for colposcopy or returning those who are negative to three- or five-yearly screening).

Previous research has focused on the effect these changes have had on the age and stage at which cervical cancer is diagnosed.^3,4^ Here we aim to describe the changes to the age and intervals at which women in the general population receive a cytology test.

## Methods

### Population

Women selected as controls in the Audit of Invasive Cervical Cancer were eligible for inclusion in this study. The Audit of Invasive Cervical Cancer is a population-based case–control study which includes screening histories from women diagnosed with cervical cancer in England between April 2007 and March 2018 and two age- and area-matched controls. The controls were matched to women with cervical cancer on age at diagnosis and place of residence. The first control had the same general practice (GP) as the case and the second had a different GP in the same geographical area. The data presented here are from the November 2019 data set released for research purposes by the PHE Office for Data Release. Data were cleaned and processed by the authors prior to analysis.

Eligible women were identified and their screening histories extracted from the cervical screening programme call–recall register. The register includes all women registered with a GP in England. Each woman’s screening history is available up to the age at which her matched case was diagnosed; therefore, not all women have the same follow-up time or calendar period when testing occurred. Women born before 1928 were excluded as they would have been over 60 years of age in 1988 when screening was introduced. Tests taken prior to 1980 were excluded because the screening call and recall computer system did not come into full effect until the national programme started in 1988. The data set includes the following data fields: year of birth, date and result of each cytology test. Broadly, test results are reported as inadequate, negative, borderline changes and low-grade dyskaryosis (Atypical squamous cells of undetermined significance [ASCUS]/ Low-grade squamous intraepithelial lesion [LSIL]), and High-grade squamous intraepithelial lesion (HSIL). Every test result is accompanied by an ‘action code’ which indicates the recommended course of action given the test result. The action codes are either routine recall (next test in 3/5 years), early recall (next test in 3, 6 or 12 months), no change (identifies tests taken outside of the programme which have no impact on programme intervals) or suspend (referral to colposcopy or test taken during follow-up). It is not possible to distinguish tests in the data set taken as a result of symptoms. However, in England tests taken in between screening rounds (i.e. without an invitation) are discouraged and often will not be processed by the laboratory.

Cervical screening in England during the study period was cytology based with triage of low-grade cytology using HPV testing from 2012/2013. Prior to HPV triage women with borderline changes and low-grade dyskaryosis were recalled at 6 or 12 months (or occasionally three months according to local practice).

### Statistics

Summary statistics were presented graphically and in tabular form by birth cohort and age at (or time since) last cytology test. The three main analyses presented in this study are detailed below. Cytology tests with an inadequate test result and those with an action code of no change are excluded from all analyses.

#### Estimating proportions tested since or prior to a given age

We calculated the proportion of women tested since age 19 using the Kaplan–Meier estimator with age as the time scale. Women exited the analysis at the time of their first adequate test (any test result, except for inadequate) or at the end of follow-up (i.e. censored at date of diagnosis of their matched case). Survival probabilities were calculated separately by year of birth. In addition, the proportion of tests taken at each year of age between age 18 and 32 by calendar year was plotted to illustrate changes to the proportion of tests taken in each single year of age over time.

The same method was used to calculate the proportion of women tested prior to age 66. Survival time (*t*) was measured backwards from age 66 to the first adequate test prior to age 66. Survival time is related to screening age (A) by *t* = 66-A.

#### Years since previous test

In this analysis first we identified adequate cytology tests taken within the age groups of interest (index test) and looked backwards in time to see if there was a previous test recorded. Number of years between tests was calculated. Women with no previous test were grouped together and shown as n/a on the graphs. Women may have contributed multiple times to this analysis. Index tests were grouped for ages 23–27, 28, 29–49, 50–52 and 53–64. The time between tests was categorised as 1, 2, 3, 4, 5, 6, 7, 8, 9, ≥10 years. Results are presented in a histogram by calendar year in which the index test was taken.

#### Interval following a negative test

To explore usual interval between tests, the time between a negative test (which resulted in a routine recall action code) and the next test was calculated using the Kaplan–Meier estimator.

We do not have information on the date of the last invitation to screening. Hence it is not possible to specifically identify non-attenders to screening. Instead, to explore the average interval between tests we estimate, for women identified as having a routine negative test, the probability of being tested again. To avoid oversampling women who attend very frequently we only considered one negative test (at random) per women in any seven-year age window. Women exited the analysis at the time of their next test (any test result, except for inadequate) or at the end of follow-up (i.e. censored at date of diagnosis of their matched case). Proportions attending testing were calculated for the following time intervals: <2.75, 2.75–3.49, 3.5–4.49 and 5.5–9.99. Results are presented separately for tests that occurred prior to 2004 and those after 2004 to account for the change in screening policy related to the length of intervals between tests.

Additionally we explored the likelihood of attending testing following a negative test among those overdue screening by 5.5 years in those aged 24.5–49  and by 7.5 years in those aged 50–59  (Supplemental material), such that: $\frac{F\left(t\right)f\mathrm{or}\;t\;>\;5.5}{1-F\left(5.5y\mathrm{rs}\right)}$ .

## Results

**Figure 1.**
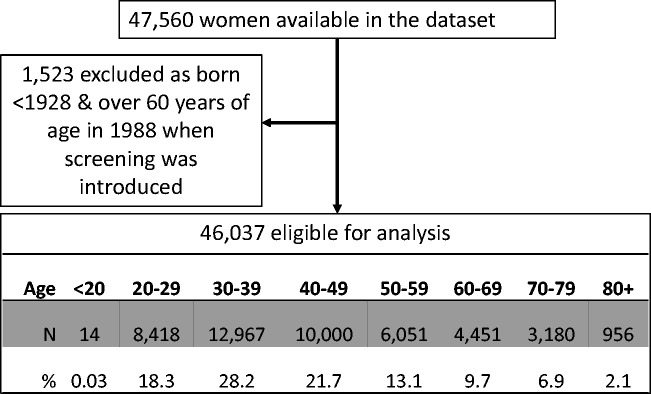
Number and age at the end of follow-up of women eligible for analysis.

Data were available for 47,560 women. We excluded 1523 women born before 1928, and thus the final data set comprised of 46,037 women. The age distribution of women included in this study reflects the age distribution of cases of cervical cancer diagnosed in England, Figure 1. The majority of women were aged 30–39  (28.2%) or 40–49 years (21.7%) at the end of follow-up, and only 9.0% were aged 70 and over (partially because those born prior to 1928 were excluded). A total of 6521 (14.2%) women have no adequate test recorded. Younger cohorts are more likely to have no cytology test recorded due to a combination of a short follow-up time and not attaining the age of 25, supplementary Table S1.

### Probability of being tested between ages 19 and 30

**Figure 2.**
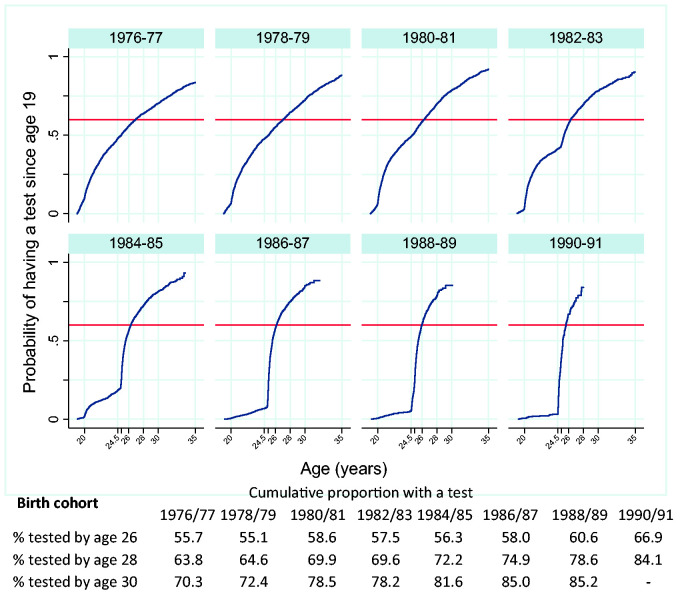
Cumulative proportion of women with a test between the ages of 19 and 35 by year of birth. Legend: vertical lines at ages 20, 24.5, 26, 28 and 31 years; horizontal line at 60%.

The effect of the age at which women are first invited for screening on attendance by age 30 years can be observed in Figure 2. The distribution of age of first test changed from a gradual increase from age 20 to 29 for birth cohorts between 1976 and 1981 to a steep drop in attendance prior to age 25 for cohorts born after 1984. Cohorts born between 1986 and 1991 have very low rates of testing under age 25 and a noticeable shift in attendance from age 25 to 24. These changes in attendance coincide with the changes to the age at which women are invited for screening.

The proportion of women tested by age 26 has increased from 55% among birth cohorts 1978–1979 to 67% among cohorts born 1990–1991, despite more recent cohorts only having received one invitation (instead of two) prior to age 26, Figure 2.

The proportion of tests taken at each single year of age between age 20 and 30 are similar between the years 1995 and 2004, Figure 3. Note the decline in tests under age 20 years around 2001 when tests at this age began to be discouraged. However, one can still see an increased proportion of tests being taken at age 23 (chequered bar) between 2001 and 2004 denoting three-yearly screening of those first invited at age 20.

**Figure 3.**
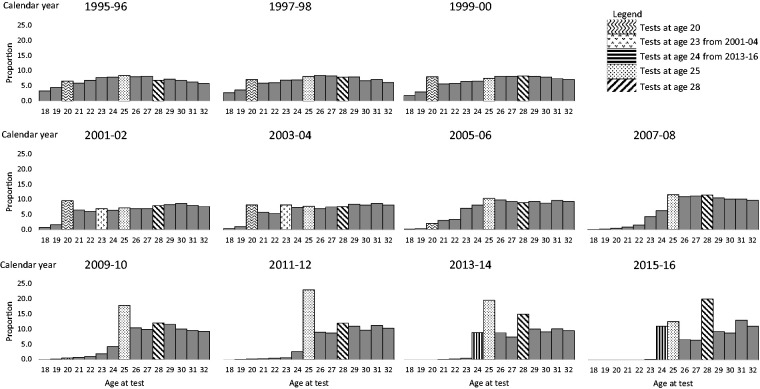
Proportion of tests taken between ages 18 and 32 by calendar year and age at test. (Production: fig shown is B&W version for print).

Between 2005 and 2008, the proportion of women tested between aged 20 and 24 decreased significantly, but the proportion tested between ages 25 and 30 remained similar. Between 2009 and 2012, the increase in tests at age 25 (dotted bar) is evident (reaching 23.0% in 2011–2012). By 2012, (three years after the first cohort of women were invited at age 25) we see another peak in tests at age 28 (12%). The only other period in which 12% of women aged 18–32 were screened at age 28 (diagonal line pattern) was between 2007 and 2010, a similar but less pronounced increase is observed among tests taken age 26, 27 and 29. These increases are most likely related to the media attention surrounding the diagnosis (August 2008) and death (March 2009) of reality TV star Jade Goody.^5^

Impact of inviting women for screening six months prior to their 25th birthday is apparent from the steady increase in tests at age 24 (vertical line pattern). By 2015–2016, 11.1% of women aged 18–32 are tested at age 24, 12.6% at age 25 and a further 20% at age 28. Also note the increase at age 31, three years later.

### Effect of standardising screening intervals nationally

**Figure 4.**
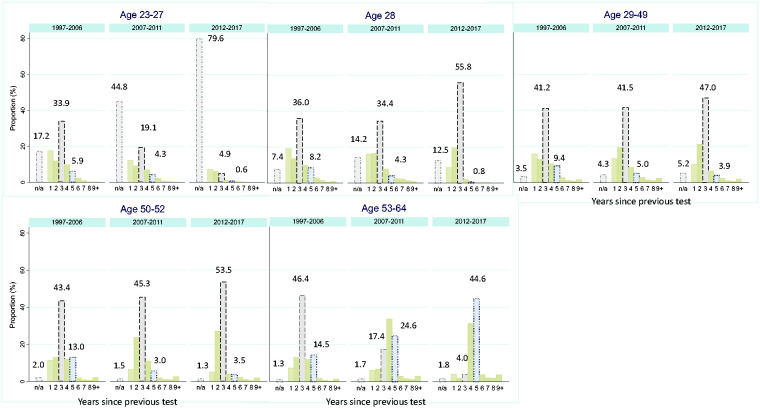
Years since previous test among women tested at ages 23–27, 28, 29–49, 50–52 and 53–64 by calendar year of and time since previous test. Legend: women with no previous tests are plotted as n/a (dash-dot line); three-yearly (dashed line) and five-yearly (dotted line) intervals.

To determine the effect of standardizing guidelines on screening intervals at other ages we explore the years to previous test, Figure 4. At all ages the consistency of the interval between tests with recommended guidelines increased over time. A decrease in women tested within a year of their previous test can be seen between 2012 and 2017 corresponding to the introduction of HPV triage.

Among women tested at age 28, the proportion whose previous test was three years earlier increased from 36% to 56% by 2012–2017. The policy to invite women from age 25 at three-yearly intervals has had the greatest impact on women tested at ages 23–27. Between 1997 and 2006, 83% of women aged 23–27 had undergone a test previously but by 2012–2016 the proportion was only 20% (i.e. women with no previous test, plotted as n/a, increased to 80%).

Women tested aged 50–52 attend after a three-yearly recall following a test in their late 40 s, whereas those tested from age 53 to 64 have seen their interval extended (slowly) to five yearly as per guidelines.

### Probability of attending next test

**Figure 5.**
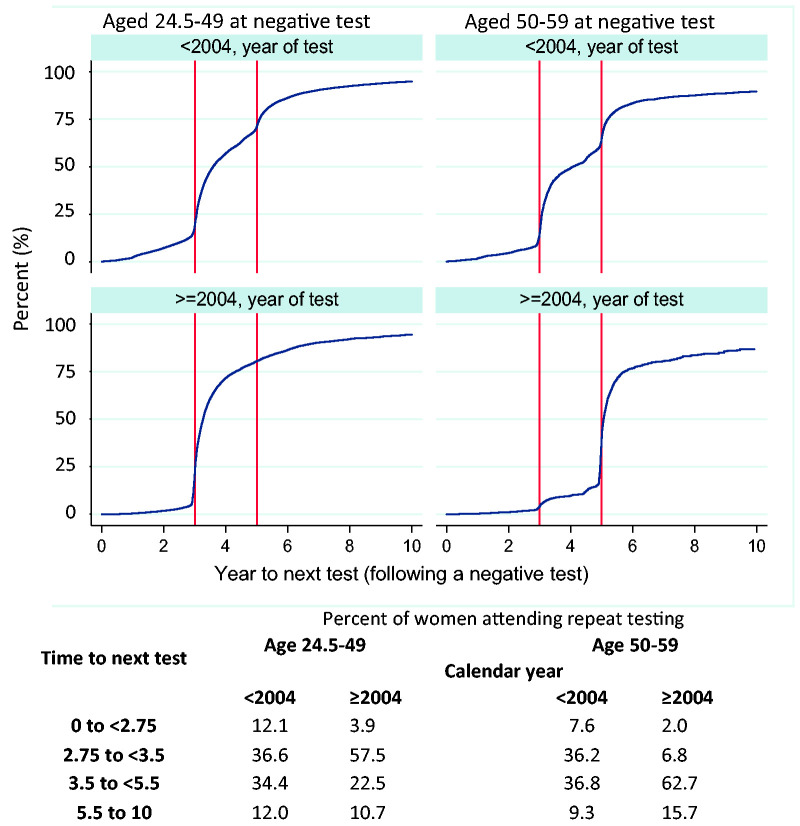
Probability that women who tested negative at least once between ages 24.5–49 and 50–59 will attend again, shown by age at negative test, calendar year of test and years between tests. Legend: vertical lines at three and five years.

Once a woman has attended testing and recieved a negative result, the probability of attending another episode within 5.5 years is high (between 71% and 84% depending on age), Figure 5. After the change in policy regarding screening intervals in 2004, an increase in women attending as recommended and a decrease in women returning at shorter intervals than recommended were observed.

Among women aged 25–49 , 58% of women with a negative test attended again as recommended. The proportion of women in this age group attending more than 3.5 years after a negative test fell from 46% prior to 2004 to 33% post 2004. Of the late attenders, most (23%) attended at an interval of 3.5–5.5 years. Only a minority (11%) show an interval of between 5.5 and 10 years.

Among women aged 50–59 post-2004, 63% attended between 3.5 and 5.5 years after a negative test, 9% attended at shorter than recommended intervals, and 16% attended at between 5.5 and 10 years. In this age group, a notable increase in women attending late was observed post-2004, Figure 5.

The probability of attending for a test among women who have not returned for 5.5 years (or 7.5 years among those aged 50 or over) decreased with age, supplementary Figure S1. Having not attended for 5.5 years, only 40% of women first tested from age 30 from 2004 onwards attended by 10 years (i.e. five years later on the graph). The equivalent figure for women first tested from age 55 who did not attend within 7.5 years was just 20%. Although attendance continues to increase upto 15 years (10 years on graph) it is not as high as that observed in Figure 5.

### Probability of being tested between ages 50 and 66

**Figure 6.**
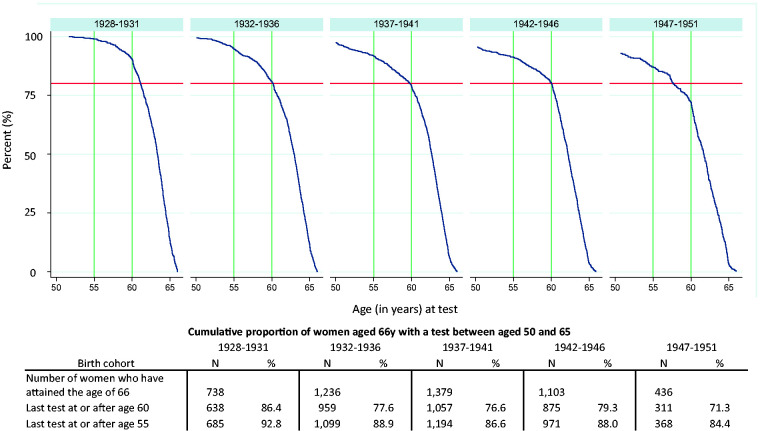
Cumulative proportion of women aged 66  with a prior test, between the ages of 50 and 65, by age at last test and year of birth. Legend: horizontal line indicates 80%, vertical lines at 55 and 60 years.

The impact of introducing five-yearly screening on the age at which women have their last test prior to exiting the programme at age 66 can be seen in Figure 6. Over 85% of women aged 66  and born between 1928 and 1931 had been tested once since age 60; this decreased to 71% among those born 1947–1951. Similarly, the percentage tested at least once after their 55th birthday decreased from 93% to 84%.

## Discussion

This is the first study to show the impact of changes to screening guidelines on intervals and ages at which women in the general population get tested. Standardisation of intervals and ages at which tests are taken can be seen within a year of screening guidelines changing, reflecting the use of IT to prospectively change screening intervals when women attend their next test as well as the systematic quality assurance arrangements in place and the maturity of the English screening programme.

Strengths of this study are that study participants represent a random sample of the population in England. Screening histories were obtained from computerised records, eliminating recall bias. We have excluded tests taken outside of the screening programme but were unable to exclude tests taken due to symptoms. National programme statistics (and this data set) report that among women eligible for screening 71% of cytology tests are either first invitations or routine recalls and a further 6% are taken following an early recall.^6^ This suggests that the results presented here reflect testing taken in a screening context.

Tests taken over the age of 65 years represent 0.68% of all cytology tests taken in England,^6^ and of these 93% are taken between ages 65 and 69. Most would have been taken in response to the final invitation to screening at age 65. By including tests up to age 66 the majority of tests taken in women over age 65 in England would have been included in the analysis.

Unfortunately due to the fact that the study participants were selected to be controls in a case–control study not all women had the same follow-up time; however, the use of survival statistics to analyse the data and stratification of results by birth cohort and calendar year should mitigate against bias due to incomplete follow-up. Due to the absence of data, it was not possible to explore attendance patterns by socio-economic and demographic characteristics of women included.

There has been concern that increasing the age of first invitation to screening would impact on the proportion of women attending screening at least once prior to age 30. It is clear that the proportion of women aged 19–34 who have had at least one cervical screening test by age 26 has not changed over time nor between birth cohorts. In fact more women have been screened by age 26 in cohorts born since 1988 despite only receiving one invitation to screening instead of two. Currently 79% of tests taken at ages 23 to 27 (not currently ages at which women get invited for screening) are taken in women who have not previously attended screening suggesting most tests at these ages are first tests in women who have delayed attending following their first invitation.

It is clear to see that inviting women for the first time to screening six months prior to their 25th birthday has resulted in a substantial proportion of them attending before or by their 25th birthday, providing evidence that this policy has achieved what it intended. Given that since the change of policy on average 65% of women have had their first test by age 26 it is not surprising that we have seen an increase in the diagnosis of cervical cancer at age 25.^3^ It also explains the increase in diagnoses of cervical cancer observed at age 24 and at age 28.^3^ In the future, we are likely to see an increase in early stage screen-detected cancer being diagnosed at specific ages corresponding to the ages at which women are invited for their repeat tests (e.g. age 28, 31, 34 etc.). A previous study reporting the impact of the diagnosis and subsequent death of reality TV star Jade Goody^5^ on attendance to screening found half a million extra cervical screening attendances (of which 370 had a test result of suspected cancer) occurred in England during this period. At its peak in March 2009, attendance was 70% higher than expected.

Importantly standardisation of screening intervals has impacted the age at which women have their last test. Whereas, previously women would have had their test closer to age 65 younger cohorts will have their last test at age 60. There is evidence to suggest that screening between ages 61 and 65 is an important predictor of risk thereafter^7^ and the period of low risk following a negative test is thought to be around 15 years.^8^ So whereas older cohorts may have been at low risk of cervical cancer up to age 80, younger cohorts may only be at low risk up to age 75. This shortening of the period at low risk may be less important once HPV primary testing is introduced into the screening programme (HPV testing has been implemented across England from December 2019).^9^ HPV testing is better than cytology at identifying those at risk of developing cervical cancer. As such it offers the opportunity for closer monitoring or for extension of screening age ranges for women who test positive between ages 60 and 65.

The introduction of HPV as a triage test for women with low-grade cytological abnormalitites has had a huge impact on screening interval, enabling women to be safely returned to routine recall testing. In this study, we can see the decrease in the proportion of tests taken at yearly intervals with the concomitant increase of three- and five-yearly testing. Recently published statistics using similar data show a halving of the proportion of women attending screening following an early recall from over 20% prior to 2010 to 10% in 2015.^10^

As has been observed in the case of breast cancer screening, once women attend screening the majority are likely to return as per guidelines. Among women who are previous attenders to breast cancer screening, uptake within five years is 82–88% (depending on age) whereas in previous non-attenders it is 36–63%.^11^ In this study 71–81% of previous attenders to cervical screening return for screening within 5.5 years whereas among women who are overdue screening for 5.5 years or more only 20–40% attend over the following five years.

Continuous monitoring of intervals and ages at which women attend screening should be a priority for the screening programme,^12^ in order to optimise its effectiveness with less screening.^13^ Going forward the most effective way to monitor the screening programme will be to carry out analyses by birth cohort. The incorporation of more sophisticated statistical analyses should provide a robust and effective way to assess the impact of introducing HPV primary screening and any changes to the screening intervals thereafter.

The results presented here give evidence that clear programme guidance and the quality assurance efforts carried out alongside have paid off, leading to swift implementation of changes to policy. Critically, among younger cohorts more women have been tested by age 26 despite only receiving one invitation to screening instead of two. Although the aim of cervical screening is to prevent cervical cancer we know that many cancers are detected at screening;^14^ hence we should expect cancer diagnoses at ages where screening invitations are due. This has implications for the age at which screen-detected cervical cancer is diagnosed and should be born in mind when interpreting incidence trends by age. The association between screening interval and average age at which women will have their last test should be considered when determining extensions to intervals in the HPV primary screening era. The screening programme has succeeded in standardising the ages and intervals at which women in England attend for screening. The remaining challenge is engaging women who do not attend screening regularly.

## Supplemental Material

sj-pdf-1-msc-10.1177_0969141320953446 - Supplemental material for Impact of changes to cervical screening guidelines on age and interval at which women are tested: Population-based study Supplemental material, sj-pdf-1-msc-10.1177_0969141320953446 for Impact of changes to cervical screening guidelines on age and interval at which women are tested: Population-based study by Alejandra Castanon, Shama Sheikh, Philippa Pearmain and Peter Sasieni in Journal of Medical Screening

## Data Availability

Data are available upon application through the PHE Office for Data Release.
