# Supplementary material for: Impact of changes to cervical screening guidelines on age and interval at which women are tested: Population-based study
Source: J Med Screen. 2020 Aug 30;28(3):325–32. doi: 10.1177/0969141320953446 (PMC8366121; doi:10.1177/0969141320953446)

Supplementary Information

This file contains supplementary table 1 and figure 1.

Table S1. Number of women eligible for analysis by age at the last cytology test and decade of birth

Decade of birth	Age at last cytology test in dataset							
	<25	25-34	35-44	45-54	55-64	65+	No tests	Total
1920*	0	0	0	2	238	131	128	499
1930	0	0	0	104	1782	587	470	2943
1940	0	3	72	319	3,025	200	366	3985
1950	1	50	313	2,532	2,226	7	307	5436
1960	51	409	4,125	3,842	90	0	579	9096
1970	281	4,861	5,642	163	0	0	1221	12168
1980	948	6,893	204	0	0	0	2882	10927
1990*	239	176	0	0	0	0	568	983
Total	1,520	12,392	10,356	6,962	7,361	925	6,521	46,037

*Note that cohort 1920 only includes 1928 and 1929. Cohort 1990 includes women up to 1996 but the majority were born in 1990 (45%), 1991(28%) or 1992 (18%)

Figure S1. Probability of attending if overdue screening by 5.5yrs in those aged 24.5-49yrs and by 7.5yrs in those aged 50-59yrs by age and year of first test and time between tests.

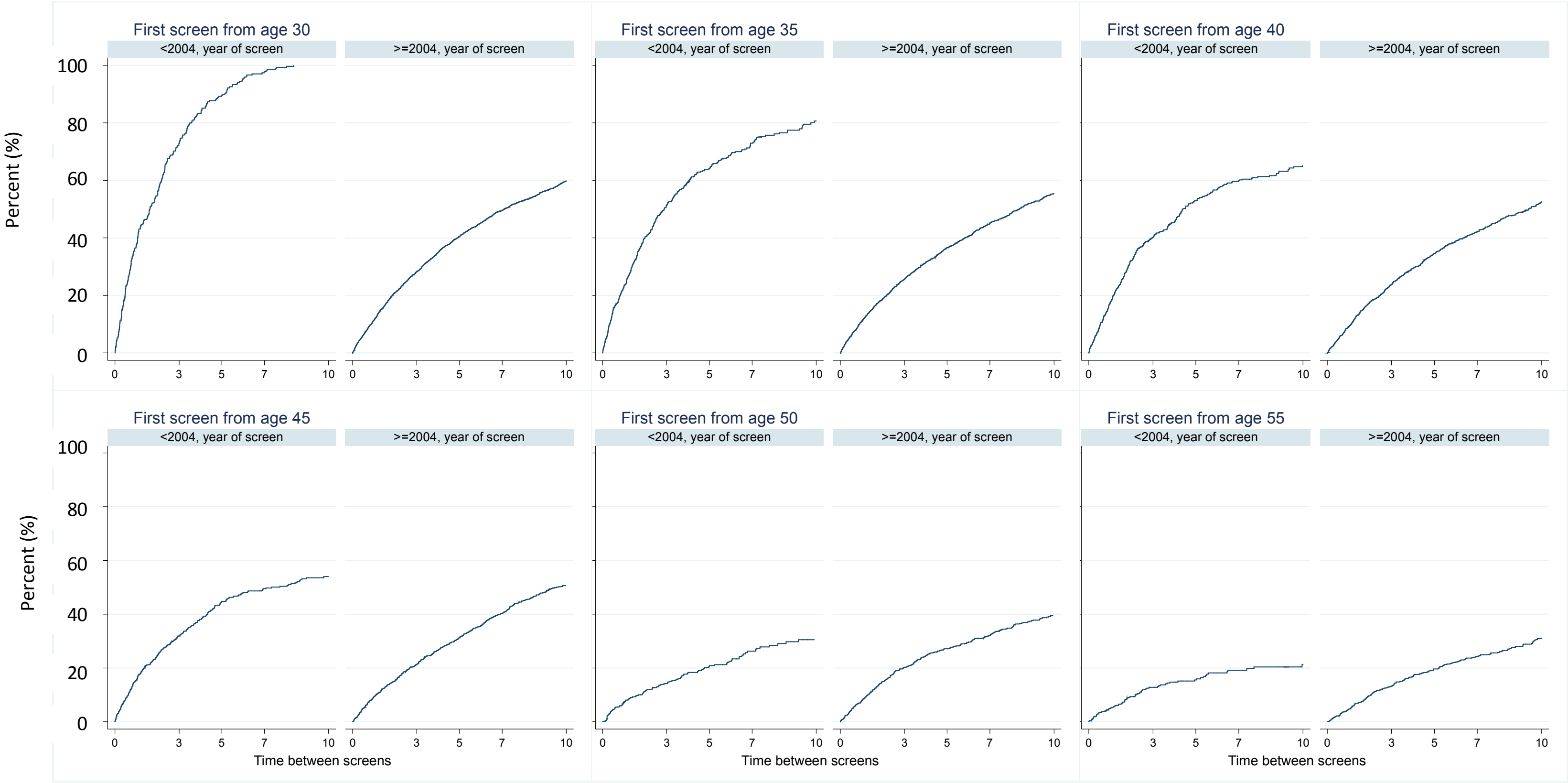

Supplement: sj-pdf-1-msc-10.1177_0969141320953446 - Supplemental material for Impact of changes to cervical screening guidelines on age and interval at which women are tested: Population-based study [file sj-pdf-1-msc-10.1177_0969141320953446.pdf]
